# Alzheimer’s disease brain-derived tau extracts show differential processing and transcriptional effects in human astrocytes

**DOI:** 10.1016/j.isci.2026.115926

**Published:** 2026-04-29

**Authors:** Matthew J. Reid, Melissa Leija Salazar, Claire Troakes, Steven Lynham, Deepak P. Srivastava, Beatriz Gomez Perez-Nievas, Wendy Noble

## Main text

(iScience *28*, 112793; July 18, 2025)

The declaration of interests for this article has been amended. Beatriz Gomez Perez-Nievas contributed to the work while employed at King’s College London. At the time of publication, she was employed by Elsevier in a role at a different journal. Peer review and editorial handling of this manuscript were conducted independently of Beatriz Gomez Perez-Nievas. The authors apologize for the oversight in the original disclosure.

